# The psychosocial outcomes of advanced hybrid closed-loop system in children and adolescents with type 1 diabetes

**DOI:** 10.1007/s00431-024-05551-1

**Published:** 2024-04-25

**Authors:** Arzu Jalilova, Birsen Şentürk Pilan, Günay Demir, Burcu Özbaran, Hanife Gul Balkı, Emrullah Arslan, Sezen Gökcen Köse, Samim Özen, Şükran Darcan, Damla Gökşen

**Affiliations:** 1 Department of Pediatric Endocrinology, Faculty of Medicine, Ege University, Izmir, Turkey; 2 Department of Child and Adolescent Psychiatry, Faculty of Medicine, Ege University, Izmir, Turkey

**Keywords:** Advanced hybrid closed-loop, Type 1 diabetes, Psychosocial outcomes, Pediatric endocrinology, Glycemic control

## Abstract

The study was carried out to determine the psychosocial outcomes of advanced hybrid closed-loop (AHCL) systems in children and adolescents with type 1 diabetes (T1D). Single-center and cohort study with a duration 6 months consisted of 60 children and adolescents with T1D. Standard clinical procedures, including both glycemic indicators, e.g., sensor-measured time within the 70–180 mg/dL range and glycated hemoglobin (HbA1c) levels, and psychosocial metrics were used for data collection. The psychosocial metrics included the Pediatric Quality of Life Inventory (PedsQL) 3.0 Diabetes Module for both children (8–12 years) and parents; the Quality of Life for Youth scale for adolescents (13–18 years); the Strengths and Difficulties Questionnaire (SDQ); the Hypoglycemia Fear Survey for Children (HFS-C); the Revised Child Anxiety and Depression Scale (R-CADS); and AHCLS-specific DTSEQ satisfaction and expectation survey. These metrics were evaluated at the baseline and after 6 months of AHCL use. Of the 60 children and adolescents with T1D for whom the AHCL system was utilized, 41 of them, 23 female and 18 male, completed the surveys. The mean age of the 41 children and adolescents was 12.5 ± 3.2 (min. 6.7, max. 18) years. The time spent within the target glycemic range, i.e., time-in-range (TIR), improved from 76.9 ± 9% at the baseline to 80.4 ± 5% after 6 months of AHCL system use (*p* = 0.03). Additionally, HbA1c levels reduced from 7.1% ± 0.7% at the baseline to 6.8% ± 0.8% after 6 months of AHCL system use (*p* = 0.03). The most notable decline in HbA1c was observed in participants with higher baseline HbA1c levels. All patients’ HFS-C and AHCL system-specific DTSEQ satisfaction and expectation survey scores were within the normal range at the baseline and remained unchanged during the follow-up period. No significant difference was found in the R-CADS scores of children and adolescents between baseline and after 6 months of AHCL system use. However, there was a significant decrease in the R-CADS scores of the parents. Patients’ PedsQL scores were high both at the baseline and after 6 months. The SDQ scores were high at baseline, and there was no significant improvement at the end of 6 months.

*  Conclusion*: This is the first study to investigate in detail the psychosocial outcomes of AHCL system use in T1D patients and their parents. Although state-of-the-art technologies such as AHCL provide patients with more flexibility in their daily lives and information about glucose fluctuations, the AHCL resulted in a TIR above the recommended target range without a change in QOL, HFS-C, SDQ, and R-CADS scores. The scores obtained from the R-CADS conducted by the parents of the children indicated that the use of pumps caused a psychological improvement in the long term, with a significant decrease in the R-CADS scores of the children and adolescents with T1D.

**Table:** 

**What is Known:** *• Previous studies focused on clinical outcomes of AHCL systems in pediatric T1D patients, showing glycemic control improvements.* *• Limited attention given to psychosocial outcomes of AHCL systems in children and adolescents with T1D.* *• Crucial psychosocial factors like quality of life, emotional well-being, and fear of hypoglycemia underexplored in AHCL system context.*	
**What is New:** *• First study to comprehensively examine psychosocial outcomes of AHCL systems in pediatric T1D patients.* *• Study's robust methodology sets new standard for diabetes technology research and its impact on qualiy of life.*

**What is Known:**

*• Previous studies focused on clinical outcomes of AHCL systems in pediatric T1D patients, showing glycemic control improvements.*

*• Limited attention given to psychosocial outcomes of AHCL systems in children and adolescents with T1D.*

*• Crucial psychosocial factors like quality of life, emotional well-being, and fear of hypoglycemia underexplored in AHCL system context.*

**What is New:**

*• First study to comprehensively examine psychosocial outcomes of AHCL systems in pediatric T1D patients.*

*• Study's robust methodology sets new standard for diabetes technology research and its impact on qualiy of life.*

## Introduction

Type 1 diabetes (T1D) is one of childhood and adolescence’s most common chronic endocrine disorders, significantly impacting their physical and emotional development [1–3]. T1D patients must make significant lifestyle adjustments to meet their daily exogenous insulin needs, including checking blood glucose levels regularly and managing dietary consumption. Difficulties in managing glycemic control negatively affect psychological health, leading to poor glycemic control and quality of life (QoL) [4, 5].

The studies indicated that automated insulin delivery (AID) systems improve glycemic control in individuals with T1D. These benefits are universally observed across different age and gender groups and are not influenced by the duration of T1D, previously used insulin delivery methods, or initial glycated hemoglobin (HbA1c) levels [6–9].

The AID systems currently available on the market require users to manually enter the carbohydrates at mealtime and indicate physical activity. Meanwhile, the system autonomously adjusts insulin delivery [10]. Compared to a number of studies on the glycemic outcomes of AID systems in this patient group, there are only a handful of studies on their psychosocial impact. Specifically, there are even fewer studies on the impact of AID systems on the QoL and emotional well-being of both children and their parents, including the quality and duration of sleep. Although the patient-reported outcomes on the impact of AID systems on QoL vary, the overarching consensus is that AID systems often improve QoL [11–13]. Additionally, some studies have reported a significant reduction in the fear of hypoglycemia [11, 13], while others have reported a decrease in diabetes-related stress and an improvement in sleep quality with the use of AID systems [14, 15].

It is important to note that advanced hybrid closed-loop (AHCL) systems do not fully automate every aspect of diabetes management. Therefore, human and psychosocial factors continue to play a significant role in effectively utilizing these systems. In this context, the objective of this study is to describe the experience of children with T1D on Minimed 780G systems in terms of diabetes difficulties, depression, anxiety, fear of hypoglycemia, and QoL.

## Materials and methods

This study was conducted as a single-center cohort study over a period of 6 months in a university outpatient clinic with a diabetes team experienced in diabetes technologies in children and adolescents with T1D. The study clinic has an average of 700 T1D children and adolescents under follow-up, encompassing various treatment modalities (40% on pump (half on hybrid closed loop) therapy 60% on MDI). The annual incidence of new onset T1D diabetes is 50.

The population of this prospective interventional study consisted of children and adolescents aged 6–18 years who used MiniMed™ 780 G (Medtronic, Northridge, CA, USA) and their parents or caregivers.

The AHCL system utilized in the country during the study period was the Minimed 780G. All patients who initiated the Minimed 780G system and completed 6 months of pump therapy were invited to participate in the study. Children and adolescents with T1D who consented to take part in the study were included irrespective of their metabolic control status.

Insulin infusion pump systems are installed in the outpatient clinic regardless of the prior treatment regimen. For patients transitioning from MDI without sensors to Minimed 780G treatment, a 10-day training process is utilized. Initially, Minimed 780G is started without sensors. On the 3rd day, sensor application and training are provided, and for patients who do not experience any issues and are able to use the sensor, automode is activated on sensor change day. The automode will start in the next 48 h. The training process for patients using sensors with MDI or other pump users is completed in an average of 7 days.

The study protocol was approved by the Ege University Ethics Committee (Approval No. 21-3.1T/23). Participants and their parents or caregivers were briefed on the objectives and methodology of the study. Informed consent was obtained from each patient included in the study.

### Study protocol

Participants were first given a tutorial in order to familiarize themselves with the manual mode of the system and its various functionalities. Auto-Mode was activated 3 and 10 days after the tutorial in those using the Minimed 640G and MDI, respectively. Pediatric psychiatrists assessed all T1D patients using the Schedule for Affective Disorders and Schizophrenia for School-Age Children/Present and Lifetime Turkish Version (K-SADS-PL-T) [16] based on the Diagnostic and Statistical Manual of Mental Disorders-5 (DSM-5) criteria [17, 18] to rule out psychiatric conditions.

All subjects, along with their parents or caregivers, were asked to fill out several validated questionnaires including the Pediatric Quality of Life Inventory (PedsQL) 3.0diabetes module [19–21], Strengths and Difficulties Questionnaire (SDQ) [22, 23], Hypoglycemia Fear Survey for Children [24, 25], The Revised Child Anxiety and Depression Scale (R-CADS) [26, 27], and the AHCL system-specific DTSEQ satisfaction and expectation survey. The questionnaires were previously translated and validated for use with Turkish children and adolescents. These assessments were completed before the use of the AID system at the baseline and after 6 months of AHCL system use.

The SDQ focuses on both positive attributes and challenges in children, consisting of items that evaluate prosocial behavior and various difficulties [23]. SDQ is divided into five categories: “conduct problems,” “hyperactivity and inattention,” “emotional symptoms,” “peer problems,” and “prosocial behaviors.” The aggregation of scores in different subsections enables the calculation of “internalizing” and “externalizing” problem indices [22]. Scoring tiers for the SDQ are categorized as normal (< 15), subclinical (15, 16), and clinical (> 16) psychosocial functioning.

The R-CADS is used for the self-assessment of anxiety and depression symptoms in children and adolescents. R-CADS consists of 47 items in six subscales, addressing various forms of anxiety and depression disorders, such as separation anxiety and social phobia. R-CADS-P, the version of R-CADS for use by parents, evaluates anxiety and depression symptoms in children and adolescents based on parental observations [28].

The Hypoglycemia Fear Survey for Children (HFS-C) comprises 24 items in behavior and worriedness subscales and additional items related to special situations related to hypoglycemia. Items are scored on a scale from 0 (never) to 4 (almost always). The total score obtained from HFS-C ranges from 24 to 120 [24].

The PedsQL 3.0 diabetes module aims to assess Health-Related QoL (HRQOL) specific to diabetes. PedsQL comes in two versions based on age groups, comprising five subscales that cover various dimensions of diabetes care, including diabetes symptoms, treatment barriers, and treatment adherence [19–21].

The DTSEQ satisfaction and expectation survey was specifically formulated for use with the “Minimed 780 G” system, utilizing the “Davis Technique” for content validity assurance [29]. The total score that can be obtained from the AHCL system-specific DTSEQ satisfaction and expectation survey ranges from 21 to 105. For content validity, a panel of 11 experts was formed, consisting of 2 pediatric endocrinology faculty members, 5 pediatric endocrinology specialists, 2 pediatric mental health faculty members, 1 pediatric mental health specialist, and 1 Ph.D nutrition specialist. The evaluations of the 11 experts were combined into a single form, and the Content Validity Index (CVI) was calculated for each item. In our assessment, the CVI was found to be 0.97, which exceeds the minimum criterion of 0.59 established by Veneziano and Hooper (1997) at the significance level of alpha 0.05 for 11 experts [30]. Thus, it can be concluded that the remaining items on the scale have been statistically evaluated for content validity.

### Statistical analysis

SPSS 22.0 (Statistical Product and Service Solutions for Windows, Version 22.0, IBM Corp., Armonk, NY, USA, 2013) software package was used to conduct the statistical analyses of the collected data. The normal distribution characteristics of the variables on all metrics related to system usage, glycemic control, and psychosocial factors were analyzed using the Kolmogorov-Smirnov test. Additionally, the Shapiro-Wilk test was used to confirm the normal distribution assumptions of quantitative data within each subset of participants. Significance in statistical variation was ascertained through multiple tests. Accordingly, the student’s *t*-test and the Mann-Whitney U test were used for normally and non-normally distributed numerical data, respectively. On the other hand, cross-tabulation, Pearson’s chi-square test, and Fisher’s exact test were used to compare the categorical data. Probability (*p*) statistics of ≤ 0.05 were deemed to indicate statistical significance. Pearson’s correlation coefficient was used to assess the correlations between normally distributed data, whereas Spearman’s rank correlation coefficient was used to assess the correlations between nonparametric data.

## Results

Of the 60 children and adolescents with T1D, for whom the AHCL system was utilized, one patient was excluded from the study due to a psychiatric diagnosis at the beginning, and additionally, 13 participants declined to participate at the beginning of the study due to their reluctance to complete the required survey assessment forms. Forty-six (67%) patients completed the 6th-month follow-up. However, four of these patients refused to fill out the sixth-month survey. In the end, 41 patients, 23 female and 18 male, were included in the study sample. The mean age was 12.5 ± 3.2 years. The mean duration of T1D was 5.5 ± 5.0 years. None of the children was on psychotropic medication during the study period. Of the 41 patients, 21 were on the Minimed 640G system, 19 on multiple daily insulin therapy (MDI), and 1 on the MiniMed Paradigm Veo pump (Table 1). Time-in-range (TIR) (70–180 mg/dL) increased from 76.9 ± 9% at the baseline to 80.4 ± 5% after 6 months of AHCL system use (*p* = 0.03). Mean HbA1c decreased from 7.1% ± 0.7% at the baseline to 6.8% ±0.8% after 6 months of AHCL system use (*p* = 0.03).

### SDQ scores

The mean total SDQ score was 24 at baseline and 22 after 6 months of AHCL system use (Table 2). There was no significant difference between baseline and sixth-month total SDQ and SDQ subscale scores.

**Table 1:** Demographic information of the participants

Age (y)	12.5 years ± 3.2
Gender (male/female)	18/23
Diabetes duration (y)	5.5 ± 5.0
Basal HbA1c % mmol/mol	7.1% ± 0.7% 54
Therapy at baseline	
Minimed VEO pump	1
Minimed 640 pump	21
MDI	19

**Table 2:** Comparison of SDQ outcomes between baseline and 6th month

SDQ (children and parents)	Baseline			6th month			
	Median	Minimum	Maximum	Median	Minimum	Maximum	***p***
***Children***							
Emotional symptoms	2	0	8	1	0	8	***0.50***
Hyperactivity/inattention	6	2	9	5	2	9	***0.06***
Conduct problems	2	0	8	2	0	7	***0.30***
Peer relationship problems	5	3	6	4	2	10	***0.30***
Prosocial behavior	9	5	10	8	5	10	***0.60***
**Total difficulty score**	24	13	30	22	13	40	***0.25***
***Parents***							
Emotional symptoms	1	0	8	2	0	7	***0.45***
Hyperactivity/inattention	5	0	13	4	0	9	***0.10***
Conduct problems	2	0	8	2	0	4	***0.08***
Peer relationship problems	5	0	12	5	0	8	***0.40***
Prosocial behavior	9	2	12	9	2	10	***0.10***
**Total difficulty score**	21	4	52	22	4	30	***0.25***

### R-CADS and R-CADS-P scores

There was no significant difference between baseline and sixth-month R-CADS scores. On the other hand, according to parents’ R-CADS-P scores, the total depression and anxiety scores of the children and adolescents decreased at the sixth month compared to baseline (Table 3).

**Table 3:** Comparison of R-CADS (children and parents) outcomes between baseline and 6th month

R-CADS (children and parents)	Baseline			6th month			
	Median	Min	Max	Median	Min	Max	***p***
***Children***							
Total anxiety score	39	27	76	39	27	80	***0.50***
Major depression scale	40	26	74	40	27	80	***0.30***
***Parents***							
Total anxiety score	47	35	80	44	34	80	***0.04***
Major depression scale	47	34	80	45	34	80	***0.03***

### PedsQL 3.0 Diabetes Module scores

There was no significant difference between baseline and sixth-month data in PedsQL3.0 Diabetes Module scores (*p* = 0.26) (Tables 4 and 5).

**Table 4:** Pediatric Quality of Life Inventory (PedsQL) 3.0 Diabetes Module outcomes between baseline and 6th month

PedsQL (child and parents)	Baseline			6th month			
	Median	Min	Max	Median	Min	Max	***p***
***Children***							
Diabetes symptoms	35	22	44	32	17	41	*0.65*
Treatment barriers	10	1	16	9.5	1	16	*0.40*
Treatment adherence	24	8	28	19	5	24	*0.30*
Worry	11	0	12	9	0	12	*0.20*
Communication	11	0	12	1	0	11	*0.10*
**Total score**	89	44	112	83	37	108	*0.25*
***Parents***							
Diabetes symptoms	35.5	16	43	35.5	13	42	*0.95*
Treatment barriers	12	3	16	11.5	2	16	*0.60*
Treatment adherence	21.5	12	28	20	4	28	*0.40*
Worry	10.5	2	12	10	4	12	*0.40*
Communication	1	0	11	9.5	5	12	*0.50*
**Total score**	87	48	108	85	37	108	*0.80*

**Table 5:** Diabetes-related quality of life for youth short form outcomes between baseline and 6th month

DQOLY-SF (adolescent)	Baseline			6th month			
	Median	Min	Max	Median	Min	Max	***p***
***Children***							
Impact of symptom (≤ 3)	2	0	5	3	0	6	*0.20*
Impact of treatment (≤ 3)	1	0	11	2	0	9	*0.45*
Impact on activities (≤ 5)	1	0	16	1	0	12	*0.15*
Parents’ issues (≤ 3)	5	0	11	6	0	9	*0.20*
Worries (≤ 7)	4	0	23	3	0	12	*0.60*
Health perception	1	0	2	1	0	2	*0.40*
**Total score**	16	0	68	17	4	43	*0.70*

### HFS-C scores

The median total HFS-C score decreased from 40 (min. 24, max. 102) at the baseline to 38 (min. 16, max. 90) at the sixth month (*p* = 0.56) (Table 6).

**Table 6:** Hypoglycemia Fear Scale for children outcomes between baseline and 6th month

HFS (child)	Baseline			6th month			
	Median	Min	Max	Median	Min	Max	***p***
***Children***							
Specific situations	11	7	29	10	6	26	*0.10*
General fears	18	9	44	18	4	40	*0.60*
Behavior	10	8	29	10	6	35	*0.95*
**Total score**	40	24	102	38	16	90	*0.55*

### AHCL system-specific DTSEQ satisfaction and expectation survey scores

The AHCL system-specific DTSEQ satisfaction and expectation survey scores were within the typical range at the baseline. There was an increase, albeit insignificant, in the total score in the sixth month (baseline mean total score: 75 ± 13 vs. sixth month mean total score: 79 ± 12; *p* = 0.16).

## Discussion

A number of studies revealed that the use of MiniMed™ 780 G (Medtronic, Northridge, CA, USA) in children with T1D aged 8 to 18 significantly increased TIR towards the target range of 70–180 mg/dL [31–33]. However, this is the first study to date addressing the long-term psychological impact of the MiniMed™ 780 G in detail. Consequently, it was determined that the anxiety and depression levels of children and adolescents included in the study significantly decreased with the use of AHCL systems, according to their parents and caregivers.

The studies on the psychosocial problems of children with T1D based on SDQ subscale scores revealed emotional and behavioral problems as their primary problems [34–36]. In contrast, a study conducted with Danish children and adolescents reported lower or comparable levels of emotional difficulties with the use of AHCL systems compared to the control group, whereas parents and caregivers of these children and adolescents reported even less favorable outcomes [36]. In a controlled study by Boogerd et al. based on SDQ scores, both children with T1D and their parents reported significantly more prosocial problems compared to the control group [37]. In contrast, a study reported significant improvements in satisfaction with the use of HCL Minimed™ 670G [38]. The improvement in outcomes with the use of HCL Minimed™ 670G can be due to reduced worriedness, increased confidence, and trust in the system with improved glycemic control. In comparison, in this study, the baseline SDQ scores revealed high total difficulty scores, whereas the SDQ subscale scores indicated symptoms of hyperactivity/inattention and peer relationship problems. These results did not improve after 6 months of AHCL system use.

A recent review by Silina showed that adolescents with T1D and their parents were more predisposed to anxiety and depression symptoms than healthy somatic children and their parents [39]. In a 4-week study conducted with 13 young children aged 7 to 10, the insulin pump therapy was changed to an AID system. Consequently, the AID system significantly reduced the depression levels of young children as well as their parents. Children’s depression scores (CDI-2) with Tandem Control IQ did not decrease significantly compared to sensor-augmented pump therapy (SAP)at the end of 4 weeks [11]. In comparison, in this study, the total R-CADS and R-CADS subscale scores of the children and adolescents with T1D were within the normal range both at the baseline and after 6 months of AHCL system use, and there were no significant differences between the baseline and sixth-month total R-CADS and R-CADS subscale scores related to anxiety and depression. On the contrary, the sixth-month R-CADS-P scores were significantly better compared to the baseline R-CADS-P, indicating better depression and anxiety outcomes. The absence of diabetes-related depression and anxiety symptoms in children and adolescents with T1D included in this study at the baseline may be attributed to good metabolic control.

In terms of QoL, the mean total PedsQL scores of the children and adolescents with T1D included in this study were high at the baseline and did not change after 6 months of AHCL system use. Similarly, Wheeler et al. did not report any significant difference in QoL scores of T1D patients with a mean age of 23 years between the use of Minimed™ 780G, SAP, and predictive low glucose suspend pump (PLGS) therapy [12]. In contrast, Beato-Vibora PI. reported a significant improvement in QoL scores after 3 months of Minimed™ 670G use [40]. Bisio et al. [11] also reported significant improvements in every measure of QoL in children with T1D with the use of AID compared to SAP. However, in the same study, the parents of these children did not report significant improvements in QoL or the emotional well-being of their children with the use of AID. In comparison, in this study, the QoL of the patients with T1D was high at the baseline, possibly due to good metabolic control. As demonstrated in previous studies, good glycemic control leads to a good QoL [41, 42]. The cultural and social factors related to family support and the fact that 53% of the patients were using a pump before they were on a flexible nutrition model might also have contributed to our patients coping with diabetes-related problems.

The total AHCL system-specific expectation and satisfaction survey scores have improved, albeit not significantly, between the baseline and after 6 months of AHCL system use. This improvement might be due to decreased worriedness, increased confidence and trust in the system, improved glycemic control, and enhanced diabetes management. This is an important finding, given that although users find AID systems helpful in terms of daily use, they report difficulties in connecting the system. Wheeler et al. found no significant difference between AID and SAP + PLGM in terms of treatment satisfaction based on the responses of diabetic adolescents and their parents [12]. In another study evaluating the hopes and expectations of family members on AID systems, it was reported that there is an expectation that this diabetes technology will alleviate diabetes-specific anxiety and burden in individuals with diabetes and other family members and that this system can reduce daily stress and, most importantly, improve family relationships [43].

In this study, no significant difference was found between baseline and sixth-month HFS-C scores. Fear of hypoglycemia, particularly night fear, is common among T1D patients and their families. Bisio et al. reported that Tandem Control IQ reduced fear of hypoglycemia in caregivers compared to SAP therapy over 1 month [11]. Although no significant difference was found in the follow-up, the decrease in the total score may reflect the decrease in fear and anxiety of hypoglycemia. Technology alarms can also cause increased levels of extra anxiety, becoming annoying, especially at night. As a matter of fact, Barnard et al. reported that alarms were a problem for some (*n* = 13) participants, noting that they were more irritating, especially when they were causing recurring sleepless nights and other family members to wake up [44].

Although AID systems improved glycemic control in our study, the decrease observed in the anxiety and depression levels of children and adolescents with T1D under real-life conditions in the long term was only from the perspective of parents and caregivers.

Many quality of life questionnaires may not encompass the specific aspects of AID therapy, potentially leading to unexpected outcomes. For example, the improvements in sleep quality resulting from stable nighttime glucose levels and the automatic correction of omitted meal boluses by the AID system are aspects that are not adequately captured by quality of life questionnaires.

In our study, several limitations need to be acknowledged. This study was conducted with a relatively small number of patients, which may limit the generalizability of the findings. Additionally, the absence of a control group prevents us from comparing the outcomes of interest to those receiving standard care or alternative treatments. These limitations should be considered when interpreting the results and designing future studies in this area. Furthermore, the study had a relatively short evaluation period of only 6 months. It is essential to consider that the long-term effects of AID pump therapy should ideally be assessed over more extended periods and at multiple time points.

In conclusion, AHCL insulin delivery represents the latest technology in the treatment of T1D. The study fills a critical gap in the literature by focusing on the psychosocial aspects of AHCL system use in pediatric T1D patients, an area previously under explored. However, this study’s findings revealed the need for further research to determine the AID systems’ long-term psychological and physical benefits.

### Concluding remarks

To the best of our knowledge, this is the first study to date to investigate in detail the psychological impact of AHCL systems on T1D patients and their parents. Even though such technologies, i.e., Minimed™ 780G systems, give flexibility to diabetic patients in their daily living activities, they did not improve their PedsQL, HFS-C, and SDQ scores or anxiety levels. On the other hand, their TIR exceeded the advised thresholds. Parents exhibited enhanced coping abilities, which may be attributed to improved sleep quality and the capability to monitor all relevant metrics.

## Data Availability

No datasets were generated or analyzed during the current study.
